# Vitexin Mitigates *Staphylococcus aureus*-Induced Mastitis via Regulation of ROS/ER Stress/NF-*κ*B/MAPK Pathway

**DOI:** 10.1155/2022/7977433

**Published:** 2022-06-27

**Authors:** Yu Chen, Jing Yang, Zhi Huang, Baoyi Yin, Talha Umar, Cheng Yang, Xiangqian Zhang, Hongyuan Jing, Shuai Guo, Mengyao Guo, Ganzhen Deng, Changwei Qiu

**Affiliations:** ^1^Department of Clinical Veterinary Medicine, College of Veterinary Medicine, Huazhong Agricultural University, Wuhan 430070, China; ^2^Department of Clinical Veterinary Medicine, College of Veterinary Medicine, Northeast Agricultural University, Harbin 150030, China

## Abstract

Mastitis, caused by a variety of pathogenic microorganisms, seriously threatens the safety and economic benefits of the dairy industry. Vitexin, a flavone glucoside found in many plant species, has been widely reported to have antioxidant, anti-inflammatory, antiviral, anticancer, neuroprotective, and cardioprotective effects. However, few studies have explored the effect of vitexin on mastitis. This study is aimed at exploring whether the antioxidant and anti-inflammatory functions of vitexin can improve Staphylococcus aureus-induced mastitis and its possible molecular mechanism. The expression profiles of S. aureus-infected bovine mammary epithelial cells and gland tissues from the GEO data set (GSE94056 and GSE139612) were analyzed and found that DEGs were mainly involved in immune signaling pathways, apoptosis, and ER stress through GO and KEGG enrichment. Vitexin blocked the production of ROS and increased the activity of antioxidant enzymes (SOD, GSH-PX, and CAT) via activation of PPAR*γ in vivo* and *in vitro*. In addition, vitexin reduced the production of inflammatory cytokines (TNF-*α*, IL-1*β*, and IL-6) and inhibited apoptosis in MAC-T cells and mouse mammary tissues infected with Staphylococcus aureus. Moreover, vitexin decreased the expression of PDI, Ero1-L*α*, p-IRE1*α*, PERK, p-eIF2*α*, and CHOP protein but increased BiP in both mammary gland cells and tissues challenged by *S. aureus*. Western blot results also found that the phosphorylation levels of JNK, ERK, p38, and p65 were reduced in vitexin-treated tissues and cells. Vitexin inhibited the production of ROS through promoting PPAR*γ*, increased the activity of antioxidant enzymes, and reduced inflammatory cytokines and apoptosis by alleviating ER stress and inactivation MAPKs and NF-*κ*B signaling pathway. Vitexin maybe have great potential to be a preventive and therapeutic agent for mastitis.

## 1. Introduction

*Staphylococcus aureus* (*S. aureus*) is an important pathogenic microorganism that causes food-borne infections, and it is also the main pathogen that causes mastitis in ruminants [1, 2]. Milk produced by cows suffering from *S. aureus* mastitis is one of the most important sources of milk contamination, whereas raw milk and dairy products are the natural medium for the growth of *S. aureus* [3]. *S. aureus* is a common opportunistic pathogen in the farm environment. Lactating dairy cows are highly susceptible to infection and transmission of *S. aureus* due to open lactation passages and exposure to contaminants such as milking equipment. The exotoxin produced by *S. aureus* can easily cause food poisoning [4]. Once exotoxin enters the gut, the T-cell inflammatory cytokine storm is triggered in the host [5, 6]. *S. aureus* is a common pathogen of dairy cow mastitis, which seriously affects milk production and quality and causes huge economic losses to the dairy industry [7, 8]. The mechanisms by which *S. aureus* challenge the host immune response are complicated. During the colonization and invasion of *S. aureus*, white blood cells are recruited to the site of infection, and white blood cells produce a series of reactive substances (including superoxide, hydrogen peroxide, nitric oxide, and hypohalous acid), which can modify and inactivate cells large molecules, even lead to growth defects or death [9]. Oxidative stress caused by *S. aureus* produces oxidants that react with cell macromolecules [10]. The accumulation of a large number of reactive oxygen species (ROS) attacked oxidized proteins, lipids, and DNA to cause oxidative damage is a characteristic of many inflammatory diseases [11]. ROS causes endoplasmic reticulum (ER) stress, which breaks the ER redox environment and induces protein folding errors [12, 13]. Persistent ER stress can also trigger an inflammatory response through the unfolded protein response (UPR) pathway [14]. ER participates in the modification (including disulfide bonds, aminoglycosylation, and folding) of secreted proteins and transmembrane proteins. Immunoglobulin heavy chain binding protein (BiP) and protein disulfide isomerase (PDI), molecular chaperone proteins in the ER, play an important role in protein folding and oxidation [15]. Protein kinase R- (PKR-) like endoplasmic reticulum kinase (PERK) and inositol-requiring enzyme 1*α* (IRE1*α*) serve as a quality controller for protein synthesis in the endoplasmic reticulum [16–18]. Once UPR is activated in the context of ER stress, BiP will separate from PERK and IRE1*α* and cause their activation, promoting the transcription of ER stress response genes [16, 19]. PERK directly phosphorylates eIF2*α* after autophosphorylation, thereby reducing protein translation and promoting the production of C/EBP homologous protein (CHOP) [20]. Elevated CHOP plays an important role in apoptosis induced by ER stress [21]. *S. aureus* also induced a cell-mediated immune response in the mouse mammary gland, which significantly promoted the production of IFN-*γ* and increased the percentage of CD4^+^ and CD8^+^ T cells [22]. The treatment of dairy cow mastitis usually uses antibiotics including penicillin, sulfonamides, tetracyclines, and glycopeptides [23]. However, the emergence of antibiotic resistance to *S. aureus* (such as methicillin-resistant *S. aureus*) complicates the treatment [24, 25]. Natural compounds have a variety of biological activities (anti-inflammatory, antioxidant, anticancer, etc.), which is a huge treasure house for screening potential mastitis treatment drugs.

Vitexin (8-beta-D-glucopyranosyl-apigenin) is an apigenin flavonoid glycoside found in food and medicinal plants, which has a variety of pharmacological effects, including anti-inflammatory, anticancer, antioxidant, analgesic, and neuroprotective effects [26, 27]. Vitexin inhibited the recruitment of neutrophils and also regulated the transcription factors of proinflammatory mediators and reduced the expression of p-p38, p-ERK1/2, and p-JNK in LPS-induced cells [28]. Studies have reported that vitexin repressed the activation of TLR4/NF-*κ*B signaling pathway in colitis liver injury [29]. Many studies have also found that vitexin exerts anti-inflammatory effects on endothelial cell damage [30], acute lung injury [31], allergic asthma [32], and neuroinflammation [33]. Both in vitro and in vivo data show that vitexin can enhance the body's antioxidant capacity and reduce the occurrence of oxidative stress [34]. Vitexin has been proven to produce a powerful antioxidant defense by acting as an effective oxygen free radical scavenger to increase the activity of antioxidant enzymes and upregulate the protein of antioxidant reactions [35]. Based on the various biological activity properties of vitexin, we hypothesized that vitexin could improve the oxidative stress and inflammation induced by *S. aureus*.

In this study, to confirm the therapeutic effect of vitexin on mastitis stimulated by *S. aureu*s, we simulated mastitis on cow mammary epithelial cells and mouse mammary glands *in vitro* and *in vivo* and explored the possible molecular mechanisms of vitexin exerting antioxidation and anti-inflammation.

## 2. Materials and Methods

### 2.1. High Pressure Liquid Chromatography (HPLC)

HPLC was performed to measure vitexin purity. Briefly, vitexin was diluted to 0.5 mg/mL by the solution (methanol : DMSO : water = 6 : 2 : 2). 20 *μ*L vitexin was added to a SinoChrom ODS-BP chromatographic column (4.6 mm × 250 mm, 5 *μ*m). Hereafter, the 0.1% phosphate water/acetonitrile mobile phase was used to elute. The flow rate was 1.0 mL/min, and the detection wavelength was 335 nm.

### 2.2. Cell Culture and Treatment

The cow mammary epithelial cell line MAC-T was purchased from the American Type Culture Collection (ATCC, USA). The cell culture method was referred to previous studies [36]. Briefly, cells were grown in DMEM/F-12 (1 : 1) medium (Gibco, USA) supplemented with 10% fetal bovine serum (FBS; AUSGENEX, AUS), 5 *μ*g/mL insulin (Biosharp, China), 5 mmol/L L-glutamine (Biosharp, China), 1 *μ*g/mL hydrocortisone (Sigma-Aldrich, USA), and 100 U/mL penicillin and 100 *μ*g/mL streptomycin (Invitrogen, USA) at 37°C incubator with 5% CO_2_. *S. aureus* CVCC3051 strain was available at China Veterinary Culture Collection (CVCC, China), which was cultured with lysogeny broth (LB) and its colony forming unit (CFU) was calculated using the LB plate counting method. *S. aureus* was inactivated in a water bath at 85°C for 15 min and then diluted it with DMEM/F-12 medium according to CFU. MAC-T cells were seeded into a six-well plate at 5 × 10^5^ cells/well. When the degree of cell fusion reached 60%-70%, *S. aureus* was added to the four wells of the six-well plate at a multiplicity of infection (MOI) of 100. After infected for 6 h, different concentrations of vitexin were added to the four wells, respectively. The six groups are (1) Control, (2) *S. aureus*, (3) *S. aureus*+10 *μ*M vitexin, (4) *S. aureus*+20 *μ*M vitexin, (5) *S. aureus*+40 *μ*M vitexin, and (6) 40 *μ*M vitexin. After incubating for another 24 h, cell samples were harvested for RNA or protein extraction.

### 2.3. Cell Counting Kit-8 (CCK-8) Assay

The effect of vitexin on the viability of MAC-T cells was analyzed by CCK-8. Detailed methods referred to previous research [37]. In short, MAC-T cells were planted into 96-well plates at a density of 1 × 10^4^ cells/well, and then, the cells were treated with different concentrations of vitexin (0, 10, 20, and 40 *μ*M) for 24 h. 10 *μ*L CCK-8 reagent (Yeasen, Shanghai, China) was added to each well and incubated at 37°C for 4 h. The microplate reader measured the OD value at 450 nm.

### 2.4. Flow Cytometry Analysis

Apoptosis was examined with Annexin V-FITC/PI Double Stain Apoptosis Detection Kit (BestBio, China). MAC-T cells were treated with *S. aureus* and/or vitexin, and then, the cells were digested and centrifuged to collect suspended cells. 400 *μ*L 1× Annexin V binding solution was used to resuspend the cells at a concentration of approximately 1 × 10^6^ cells/mL. Then, 5 *μ*L Annexin V-FITC solution was added to the cell suspension, mixed gently, and incubated for 15 min at 4°C in the dark. Then, 5 *μ*L PI was added and incubated for 5 min at 4°C in the dark. Finally, the flow cytometer was hired immediately to obtain the results.

### 2.5. Mouse and Mastitis Animal Models

A total of 60 KM female mice (6-7 weeks old, 32–34 g weight) were gained and fed in Huazhong Agricultural University Laboratory Animal Research Center (Wuhan, China). All mice were allowed to obtain food and water ad libitum in the environment maintained at 25°C ± 1°C and 65% humidity. All procedures of this study were carried out in accordance with standards provided by the Ethical Committee on Animal Research at Huazhong Agricultural University.

All mice were randomly divided into 6 groups: control group, *S. aureus* group, *S. aureus* + vitexin (15, 30, and 60 mg/kg) groups, and vitexin (60 mg/kg) group. *S. aureus* was injected into the mammary ducts of mice to produce mastitis in mice according to previous studies [7]. Vitexin was diluted with sterile PBS to 20 mg/mL. After mouse mammary gland infection with *S. aureus* for 24 h, the control group was intraperitoneally injected with sterile PBS. As the previous study described the dosage of vitexin [38, 39], the *S. aureus* + vitexin (15, 30, and 60 mg/kg) groups were intraperitoneally injected with vitexin at different doses (15, 30, and 60 mg/kg); the vitexin (60 mg/kg) group was intraperitoneally injected with vitexin at 60 mg/kg. All groups were injected three times with an interval of 8 h. Then, the mice were euthanized at 8 h after the last drug treatment, and breast tissues were harvested and stored at −80°C refrigerator.

### 2.6. Hematoxylin and Eosin (H&E) Staining

H&E staining was used to evaluate the histopathology of *S. aureus* infection in mouse breast tissue. The experimental method was in accordance with the previous research [40]. In short, the collected fresh mouse breast tissue was immediately fixed in 4% paraformaldehyde solution, paraffin embedded, sectioned, transparent, dehydrated, and H&E stained. Finally, the histopathological changes were checked by an optical microscope.

### 2.7. Myeloperoxidase (MPO) Activity

MPO Assay Kit (Nanjing Jiancheng Bioengineering Institute, China) was bought to detect MPO activity. The mouse mammary gland tissue was accurately weighed, and the homogenization medium (*w* : *v* = 1 : 19) was added to prepare a 5% tissue homogenate.

### 2.8. Enzyme-Linked Immunosorbent Assay (ELISA)

The effect of vitexin on proinflammatory cytokine production was examined in *S. aureus* challenge-MAC-T cells and mouse breast tissues. The concentration of TNF-*α*, IL-1*β*, and IL-6 was detected by ELISA kits (BioLegend, San Diego, CA, USA) following the manufacturer's instructions.

### 2.9. ROS Detection

Intracellular ROS level was measured with DHE-ROS Detection Kit (BestBio, Shanghai, China) according to the manufacturer's manual. Briefly, MAC-T cells were pretreated for 30 min with GW9662 (10 *μ*M) or GW1929 (10 *μ*M) to downregulate or upregulate the expression of PPAR*γ*, respectively. Cells were stimulated with *S. aureus* to mimic inflammatory stimulation and then treated with vitexin (40 *μ*M) for 24 h. The DHE probe was diluted 1000 times with DMEM/F-12 medium and then added to the cells to continue to incubate in the dark for 30 min at 37°C. The cells were washed with PBS to remove the DHE probe and finally observed with a fluorescence microscope at the excitation wavelength (Ex) of 535 nm and the emission wavelength (Em) of 610 nm.

Tissue ROS Detection Kit (BBoxiProbe O13®) was obtained from BestBio (Shanghai, China). The harvested fresh mouse mammary gland tissue was accurately weighed and then prepared into a homogenizer with a glass homogenizer. The homogenate supernatant was collected by centrifugation and added into 96-well plate with 2 *μ*L probe O13. After incubating for 30 min at 37°C in the dark, the fluorescence intensity was detected with a fluorescence microplate reader at Ex/Em = 535/610 nm.

### 2.10. Antioxidant Biochemical Indicators

Total antioxidant capacity (T-AOC), superoxide dismutase (SOD), glutathione peroxidase (GSH-PX), catalase (CAT), and malondialdehyde (MDA) were determined to assess antioxidant capacity. T-AOC assay kit, SOD assay kit (WST-1 method), GSH-PX assay kit, CAT assay kit (visible light), and MDA assay kit (TBA method) were bought from Nanjing Jiancheng Bioengineering Institute (Nanjing, China), and the inspection process was carried out in accordance with the instructions.

### 2.11. Real Time-Quantitative Polymerase Chain Reaction (RT-qPCR)

The total RNA of mouse mammary gland tissue and MAC-T cells was extracted with TRIzol reagent (Solarbio, Beijing). Q5000 ultramicro spectrophotometer (Quawell, USA) was used to evaluate RNA purity and measure RNA concentration. Hifair® III 1st Strand cDNA Synthesis SuperMix for qPCR (gDNA digester plus) and Hieff® qPCR SYBR® Green Master Mix were acquired from Yeasen (Shanghai, China) and used to examine target gene expression by qPCR with 2^−*ΔΔ*Cq^ method. The primers used in this study are shown in Table 1.

### 2.12. Western Blot

The total protein of mouse breast tissues and MAC-T cells was separated by RIPA reagent (Biosharp, China). The BCA Protein Assay Kit (Thermo Scientific, MA, USA) was performed to detect protein concentration. The protein was separated by sodium dodecyl sulfate-polyacrylamide gel electrophoresis (SDS-PAGE); then, polyvinylidene difluoride (PVDF) membranes were used to receive the transferred protein from the gel. The membranes were blocked with 5% skimmed milk for 2 h; then, the primary antibodies (BiP, Ero1-L*α*, PDI, IRE1*α*, PERK, CHOP, JNK, ERK, p38, p65, and *β*-actin from Cell Signaling Technology, USA; PPAR*γ* from Abcam, UK) were incubated at 4°C overnight, and then, the secondary antibody (anti-rabbit IgG and HRP-linked antibody from Cell Signaling Technology, USA) was incubated at 37°C for 1 h. Finally, the immunoblot signal was displayed with ECL ultrasensitive chemiluminescent solution with chemiluminescence imaging system.

### 2.13. Bioinformatics Analysis

Expression profiles of *S. aureus*-infected bovine mammary epithelial cells and glandular cistern (GSE94056 and GSE139612) were downloaded from the GEO dataset (https://www.ncbi.nlm.nih.gov/gds/?term=). Differentially expressed genes (DEGs) were screened from these two datasets, and common DEGs were further analyzed by the online tool Draw Venn Diagrams (http://bioinformatics.psb.ugent.be/webtools/Venn/). Gene Ontology Resource (GO, http://geneontology.org/) and Kyoto Encyclopedia of Genes and Genomes (KEGG, https://www.kegg.jp/) were used to analyze GO and KEGG enrichment of common DEGs. In addition, protein-protein interaction (PPI) network was built by STRING database (https://string-db.org/).

The druggability of vitexin was assessed by the Traditional Chinese Medicine System Pharmacology Database (TCMSP, https://tcmspw.com/), and the potential targets of vitexin were identified using SwissTarget Prediction (http://www.swisstargetprediction.ch/).

### 2.14. Statistical Analysis

Statistical analysis was processed with the GraphPad Prism 9.00 software. Statistical data were expressed as mean ± SEM of three individual experiments. After the normality test, differences between groups were analyzed by one-way ANOVA or Student's *t*-tests. #*p* < 0.01 vs. the control group, ∗*p* < 0.05 vs. the *S. aureus* group, and ∗∗*p* < 0.01 vs. the *S. aureus* group.

## 3. Results

### 3.1. DEG Analysis of S. aureus-Induced Mastitis from GEO Database

**Figure 1:**
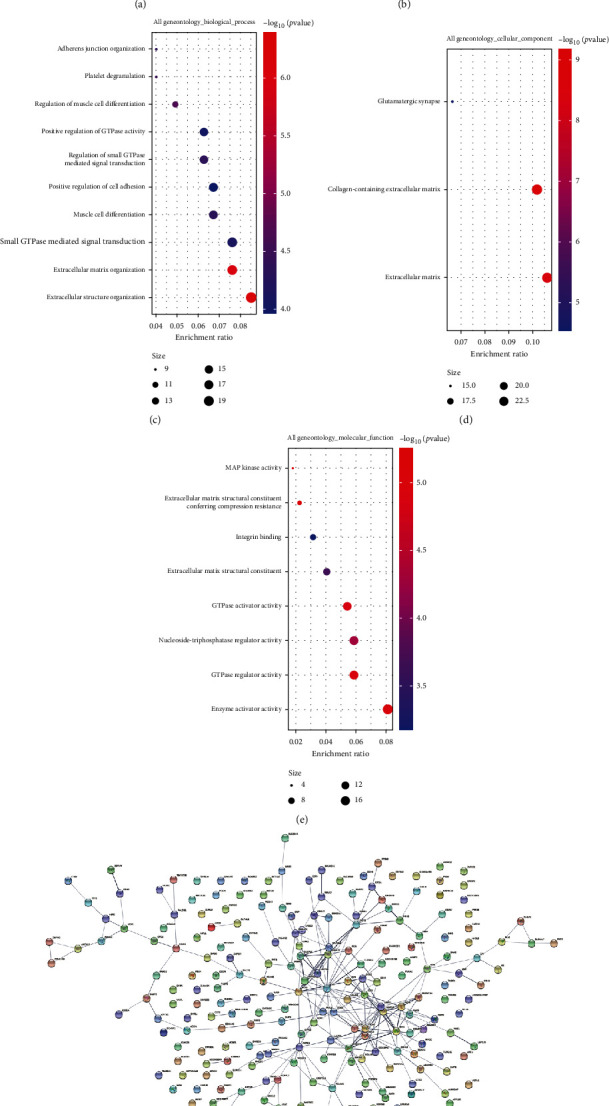
DEG analysis of *S. aureus*-induced mastitis from GEO database. (a) The Venn diagram displayed the distribution of DEGs in two GEO datasets (GSE94056 and GSE139612). (b) KEGG enriched these common DEG pathways. (c–e) GO enrichment analysis on 283 DEGs, including biological process, cellular components, and molecular functions. (f) The interaction between 283 DEGs was analyzed through the STRING database. (g) Emphasized the interaction relationship with CHOP- (DDIT3-) related DEGs.

Given that the host infection event usually undergoes a large number of complex gene expression changes, we analyzed the expression profiles of bovine mammary epithelial cell and gland cistern infected with *S. aureus* from the GEO dataset (GSE94056 and GSE139612) [41, 42]. In these two GEO datasets, GEO2R was used to analyze the DEGs [43, 44]. Furthermore, the intersection of these DEGs was calculated through drawing Venn diagrams. The DEGs of two GEO datasets contained 283 identical DEGs (Figure 1(a)). These 283 DEGs were further analyzed by KEGG and GO enrichment. We found that these DEGs mainly involve innate immunity and apoptosis signaling pathways (MAPK signaling pathway, TNF signaling pathway, Toll-like receptor signaling pathway, RIG-I-like receptor signaling pathway, etc.) (Figure 1(b)). The biological process that DEGs participated in included extracellular structure organization, extracellular matrix organization, small GTPase-mediated signal transduction, muscle cell differentiation, positive regulation of cell adhesion, regulation of small GTPase-mediated signal transduction, positive regulation of GTPase activity, regulation of muscle cell differentiation, platelet degranulation, and adherens junction organization (Figure 1(c)). These DEGs were involved in the extracellular matrix, collagen-containing extracellular matrix and glutamatergic synapses in cellular components term (Figure 1(d)). The molecular functions of these DEGs were enzyme activator activity, GTPase regulator activity, nucleoside-triphosphatase regulator activity, GTPase activator activity, extracellular matrix structural constituent, integrin binding, extracellular matrix structural constituent, conferring compression resistance, and MAP kinase activity (Figure 1(e)). To further explore the interaction between these DEGs, we used the STRING database to build an interaction network diagram (Figure 1(f)) and built an interaction network diagram for the extremely significant DEGs separately (Figure 1(g)).

### 3.2. Network Pharmacology Analysis of Vitexin

**Figure 2:**
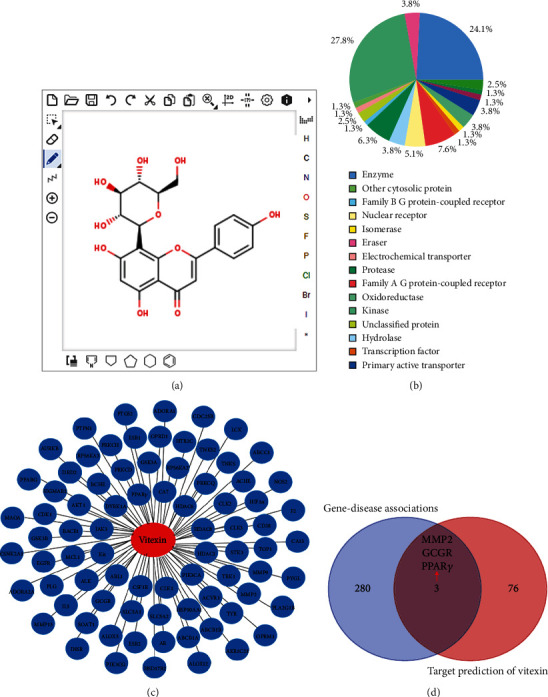
Network pharmacology analysis of vitexin. (a) The chemical structural formula of the searched molecule. (b) The target classes of vitexin. (c) The potential targets of vitexin were predicted on the SwissTarget Prediction. (d) The common target genes of vitexin and *S. aureus*-induced mastitis.

As displayed in Figure 2, our results showed that vitexin has good druggability with 79 potential target genes. There were three target genes (PPAR*γ*, MMP2, and GCGR) that intersect with common DEGs, among which PPAR*γ* was involved in oxidative stress.

### 3.3. The Purified Substance Vitexin Had No Effect on the Viability of MAC-T Cells

**Figure 3:**
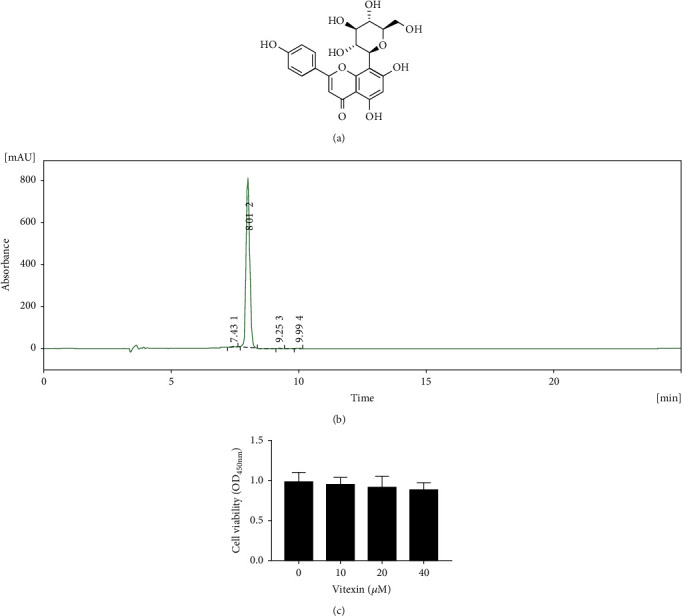
The purified substance vitexin had no effect on the viability of MAC-T cells. (a) The chemical structure of vitexin. (b) HPLC for purity inspection of vitexin. (c) CCK-8 assessed the effect of vitexin on the viability of MAC-T cells.

Vitexin is the natural 8-C glucoside of the flavone apigenin (mung bean [45], chia leaf [46], Prosopis cineraria leaves [47], etc.), which is very likely to become a natural antioxidant. As illustrated in Figure 3(a), vitexin is a flavone glucoside, also known as apigenin-8-C-*β*-D-glucopyranoside. The purity of the extracted vitexin was detected by HPLC, and the extract was found to be a high-purity vitexin (Figure 3(b)). For the follow-up *in vivo* and *in vitro* experiments of vitexin, we tested and analyzed the effect of vitexin on cytotoxicity through CCK-8. CCK-8 results showed that vitexin was not toxic to MAC-T cells at least in the concentration range below 40 *μ*M (Figure 3(c)).

### 3.4. Vitexin Ameliorated Cell Damage on *S. aureus* Challenged-MAC-T Cells

**Figure 4:**
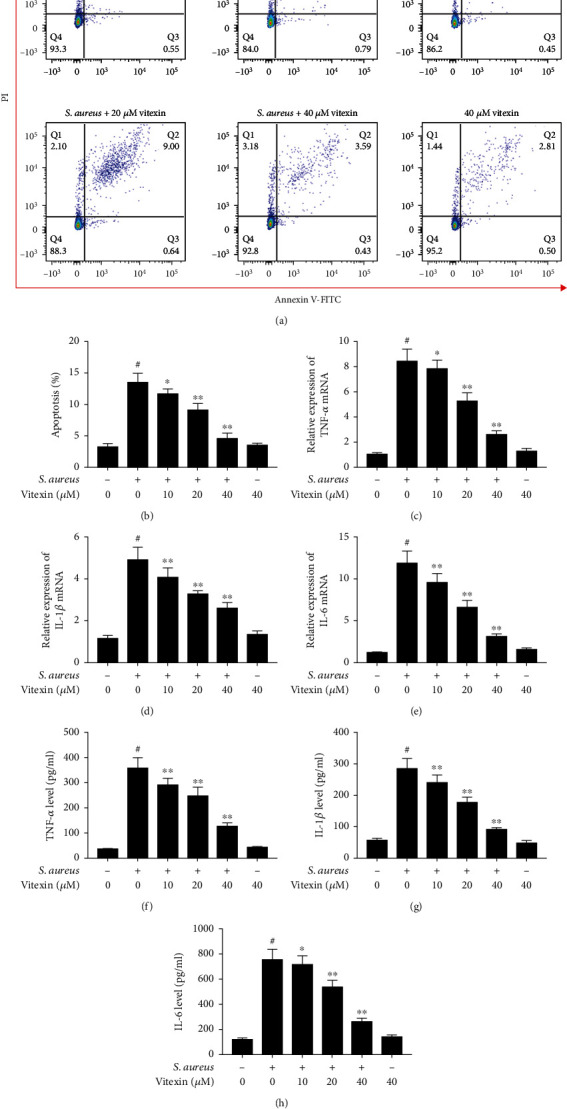
Vitexin ameliorated cell damage on *S. aureus* challenged-MAC-T cells. (a) MAC-T cells were stimulated with *S. aureus* at MOI of 100 for 6 h. And then, different concentrations of vitexin (10, 20, and 40 *μ*M) were added to coincubate for 24 h. Flow cytometry was used to evaluate the effect of vitexin on the apoptosis of MAC-T cells stimulated by *S. aureus*. The horizontal axis represented Annexin V-FITC, and the vertical axis expressed PI. (b) Flow cytometry results were statistically analyzed for apoptosis rate by the GraphPad Prism 9.00 software. (c–e) The cells were dealt with *S. aureus* and/or vitexin; the relative expression of TNF-*α*, IL-1*β*, and IL-6 mRNA was examined by RT-qPCR. GAPDH served as an internal reference gene. (f–h) The proinflammatory factors produced were tested by ELISA. Data were presented as means ± SEM of three independent experiments. #*p* < 0.01 vs. the control group. ^∗^*p* < 0.05 vs. the *S. aureus* group; ^∗∗^*p* < 0.01 vs. the *S. aureus* group.

MAC-T cells infected with *S. aureus* experienced significant apoptosis (Figure 4(a)). But vitexin reduced the apoptosis rate of *S. aureus*-infected MAC-T cells in a dose-dependent manner (Figure 4(b)). In addition, the level of proinflammatory cytokine production was evaluated in *S. aureus*-induced MAC-T cells by qPCR and ELISA. The mRNA level of proinflammatory cytokines was greatly increased under the stimulation of *S. aureus*. On the other hand, vitexin inhibited the production of inflammatory factors in a dose-dependent manner (Figures 4(c)–4(e)). The released TNF-*α*, IL-1*β*, and IL-6 concentrations in the cell supernatant were evaluated by ELISA, whose results were consistent with the trend of qPCR (Figures 4(f)–4(h)).

### 3.5. Vitexin Inhibited ROS Production on S. aureus Stimulated-MAC-T Cells

**Figure 5:**
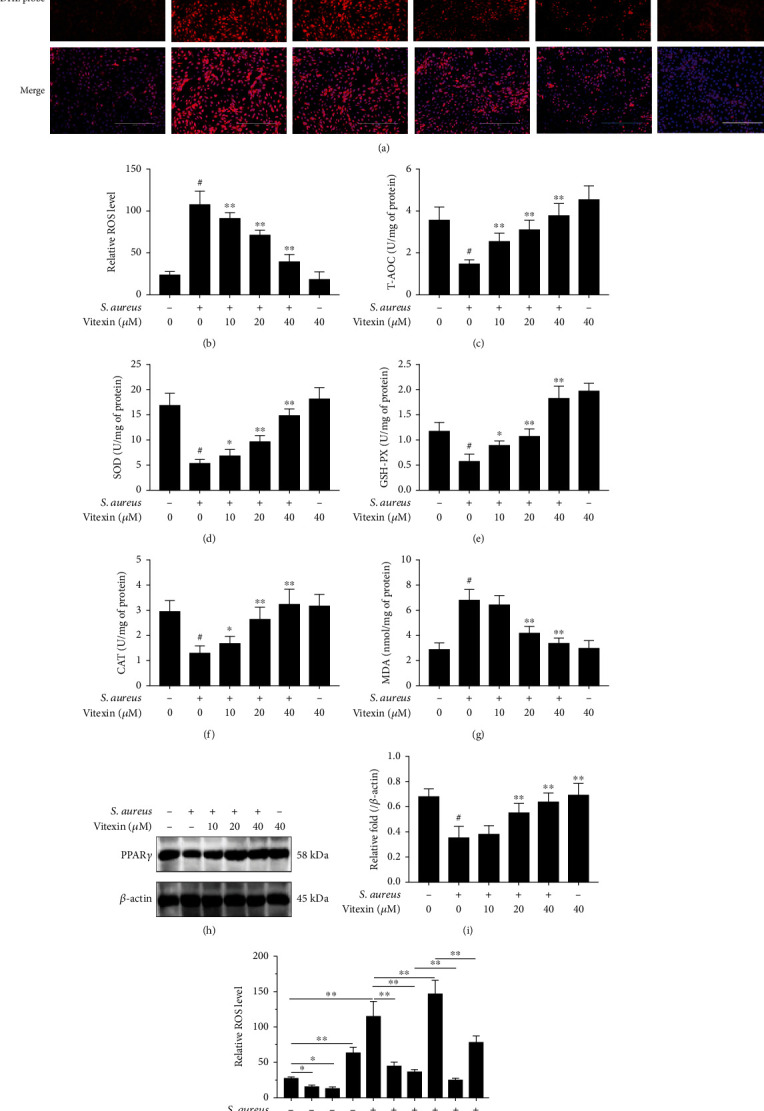
Vitexin inhibited ROS production on *S. aureus* stimulated-MAC-T cells. (a) The cells were stimulated with *S. aureus* for 6 h and then treated with different concentrations of vitexin (10, 20, and 40 *μ*M) for 24 h. ROS red fluorescence was displayed with DHE probe. (b) ROS levels were quantified by the ImageJ software. Relative ROS levels were expressed as red fluorescence intensity/cell number. (c) The level of T-AOC. (d) The activity of SOD. (e) The activity of GSH-PX. (f) The activity of CAT. (g) The concentration of MDA. (h) Western blot detection of the expression level of PPAR*γ*. (i) PPAR*γ* gray values were measured by the ImageJ software. (j) MAC-T cells were pretreated with GW9662 (10 *μ*M) or GW1929 (10 *μ*M) for 30 min to downregulate or upregulate the expression of PPAR*γ*, respectively, then stimulated with *S. aureus* to mimic inflammatory stimulation, and finally treated with vitexin. ROS was detected with DHE probe. Data was expressed as means ± SEM of three independent experiments. #*p* < 0.01 vs. the control group. ^∗^*p* < 0.05 vs. the *S. aureus* group; ^∗∗^*p* < 0.01 vs. the *S. aureus* group.

The level of intracellular ROS was evaluated by DHE probe, and the results revealed that *S. aureus* infection of MAC-T cells rapidly promoted the production of intracellular ROS. However, vitexin could effectively suppress the MAC-T cells stimulated by *S. aureus* to produce a large amount of ROS (Figures 5(a) and 5(b)). The body's antioxidant capacity is closely related to enzymes such as SOD, GSH-PX, and CAT, which eliminate free radicals and ROS so as not to trigger lipid peroxidation. We tested the T-AOC and the abovementioned enzyme activity. The T-AOC and enzyme activities of SOD, GSH-PX, and CAT in MAC-T cells stimulated by *S. aureus* were much lower than control, but vitexin obviously increased the T-AOC, SOD, GSH-PX, and CAT activities on *S. aureus* challenged-MAC-T cells (Figures 5(c)–5(f)). Conversely, vitexin prevented the production of MDA in a dose-dependent manner on MAC-T cells stimulated by *S. aureus* (Figure 5(g)). To further explore the mechanism of the antioxidant effect of vitexin, PPAR*γ* was predicted as an interacting protein by investigating the possible target genes of vitexin. Western blot results revealed that PPAR*γ* was inhibited after *S. aureus* infection, whereas vitexin greatly promoted PPAR*γ* expression (Figures 5(h) and 5(i)). Agonists (GW1929) and antagonists (GW9662) of PPAR*γ* were hired to further study the effect of vitexin on PPAR*γ*. As shown in Figure 5(j), vitexin, like PPAR*γ* antagonists, reduced ROS levels in uninfected cells. ROS levels increased sharply under S. aureus stimulation, whereas agonists significantly reduced ROS levels in *S. aureus*-stimulated cells and were less effective than vitexin and agonist combination treatment. Meanwhile, PPAR*γ* antagonists accelerated the increase of ROS in *S. aureus*-infected cells, but when antagonists were used in combination with vitexin, GW9662 prevented the ROS inhibitory effect of vitexin under *S. aureus* challenge.

### 3.6. Vitexin Suppressed the Activation of MAPK and NF-*κ*B Signaling Pathways by Blocking the ER Stress Caused by S. aureus on MAC-T Cells

**Figure 6:**
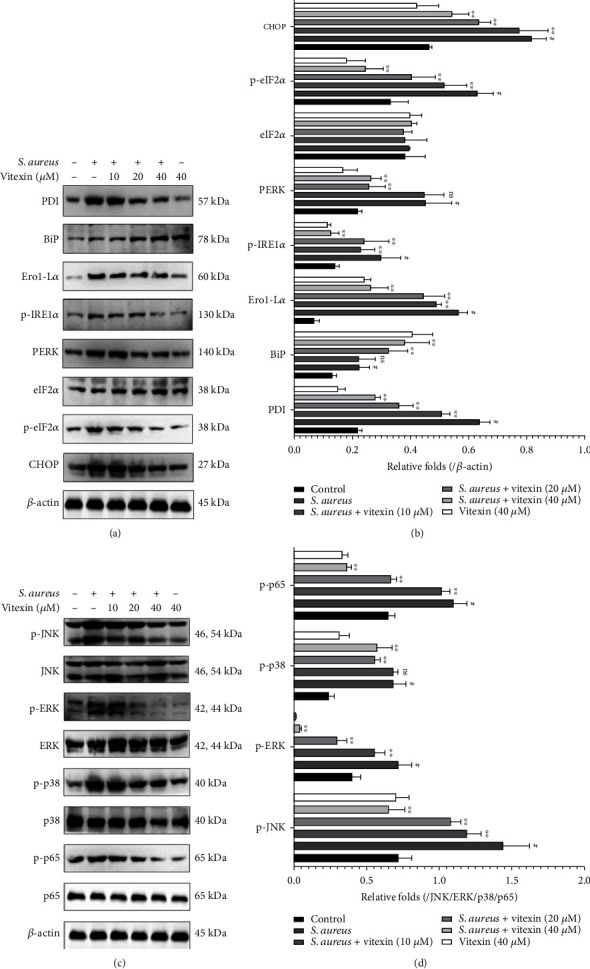
Vitexin suppressed the activation of MAPK and NF-*κ*B signaling pathways by blocking the ER stress caused by *S. aureus* on MAC-T cells. (a) The protein levels of PDI, BiP, Ero1-L*α*, p-IRE1*α*, PERK, p-eIF2*α*, and CHOP were detected by Western blot on MAC-T cells. (c) The expression levels of JNK, ERK, p38, and p65 proteins in MAC-T cells were determined by Western blot. (b, d) Protein gray values were measured by the ImageJ software. Data was expressed as means ± SEM of three independent experiments. #*p* < 0.01 vs. the control group. ^∗^*p* < 0.05 vs. the *S. aureus* group; ^∗∗^*p* < 0.01 vs. the *S. aureus* group.

To investigate the effect of vitexin on ER stress, ER stress-related proteins were examined by Western blot. The levels of BiP in *S. aureus*-infected cells were lower than normal cells, but vitexin greatly promoted their expression levels under the stimulation of *S. aureus*. Meanwhile, *S. aureus* infection drastically increased the expression of PDI, Ero1-L*α*, p-IRE1*α*, PERK, p-eIF2*α*, and CHOP, while vitexin repressed their expression (Figures 6(a) and 6(b)). In addition, Western blot was also hired to evaluate MAPK and NF-*κ*B signaling pathways. The phosphorylation levels of JNK, ERK, p38, and p65 in *S. aureus*-induced MAC-T cells dramatically increased compared to the control. Reversely, vitexin significantly restricted the expression of p-JNK, p-ERK, p-p38, and p-p65 (Figures 6(c) and 6(d)).

### 3.7. Vitexin Improved S. aureus-Induced Mastitis in Mice

**Figure 7:**
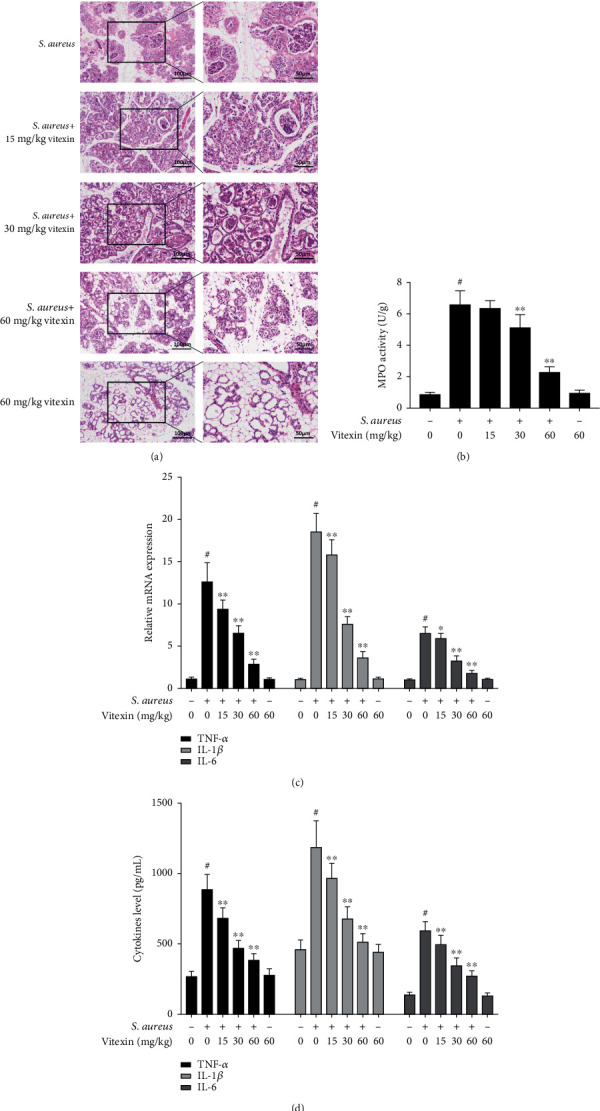
Vitexin improved *S. aureus*-induced mastitis in mice. All mouse mammary gland tissues were stimulated with *S. aureus* for 24 h, then intraperitoneally injected with different concentrations of vitexin (15, 30, and 60 mg/kg) three times. (a) Histopathological analysis of mammary gland tissues with H&E staining. Left images scale bar = 100 *μ*m, right large images scale bar = 50 *μ*m. (b) The activity of MPO. (c) The expression of TNF-*α*, IL-1*β*, and IL-6 mRNA in vivo was measured by RT-qPCR. GAPDH was used as an endogenous control. (d) The proinflammation cytokines TNF-*α*, IL-1*β*, and IL-6 in mammary gland tissues were detected by ELISA. Data was expressed as means ± SEM of three independent experiments. #*p* < 0.01 vs. the control group. ^∗^*p* < 0.05 vs. the S. aureus group; ∗∗*p* < 0.01 vs. the S. aureus group.

A mouse model of mastitis was established to assess the protective effect of vitexin. H&E staining was performed to evaluate the degree of damage to the mammary gland tissue of mice infected with *S. aureus*. As illustrated in Figure 7(a), the acinar cavity epithelial cells of mice mammary glands were attacked by *S. aureus* causing degeneration, necrosis, and even shedding to the acinar cavity. At the same time, the acinar cavity was also filled with serum and a large number of neutrophils and macrophages. Vitexin maintained the integrity of the acinar cavity epithelial cells and relieved the infiltration of inflammatory cells. Moreover, MPO was checked to evaluate the degree of inflammation in the mammary gland tissues, and the result indicated that vitexin remarkably suppressed MPO vitality in a dose-dependent manner (Figure 7(b)). In addition, qPCR and ELISA were employed to examine proinflammatory cytokines (TNF-*α*, IL-1*β*, and IL-6) which were depressed by vitexin both in mRNA and protein levels (Figures 7(c) and 7(d)).

### 3.8. Vitexin Reduced ROS Production in Mammary Tissues of Mice Infected with S. aureus

**Figure 8:**
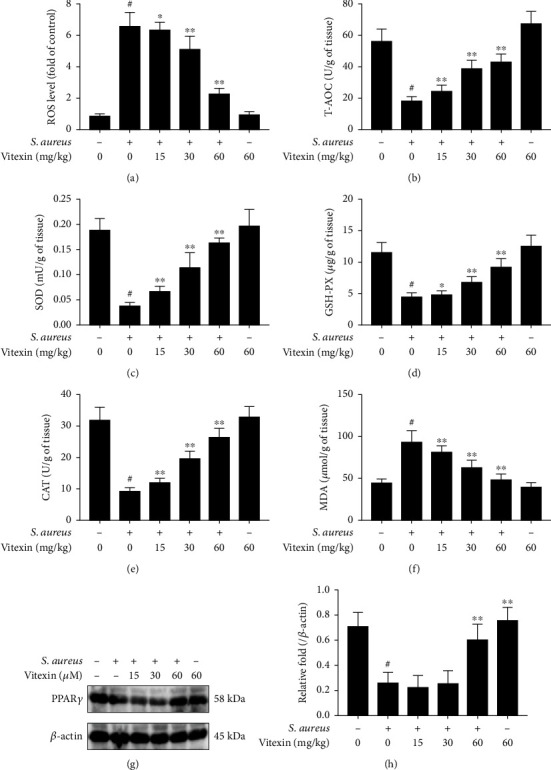
Vitexin reduced ROS production in mammary tissues of mice infected with *S. aureus*. (a) The level of tissue ROS. (b) The level of T-AOC. (c) The activity of SOD. (d) The activity of GSH-PX. (e) The activity of CAT. (f) The concentration of MDA. (g) PPAR*γ* protein was examined with Western blot. (h) PPAR*γ* gray values were calculated by the ImageJ software. Data was expressed as means ± SEM of three independent experiments. #*p* < 0.01 vs. the control group. ^∗^*p* < 0.05 vs. the *S. aureus* group; ^∗∗^*p* < 0.01 vs. the *S. aureus* group.

The tissue ROS and antioxidant capacity of mice with mastitis were determined. We found that *S. aureus* infected mouse mammary tissue strikingly increased the level of tissue ROS, whereas vitexin hindered the rise of ROS (Figure 8(a)). Furthermore, vitexin reversed *S. aureus* induced the fall of T-AOC (Figure 8(b)), SOD (Figure 8(c)), GSH-PX (Figure 8(d)), and CAT (Figure 8(e)). In contrast, MDA level was impeded by vitexin, compared with the *S. aureus* group (Figure 8(f)). PPAR*γ* protein levels were downregulated in *S. aureus*-infected cells, and vitexin reversed this decline (Figures 8(g) and 8(h)).

### 3.9. Vitexin Relieved ER Stress to Inactivate MAPK and NF-*κ*B Signaling Pathways in S. aureus-Induced Mastitis in Mice

**Figure 9:**
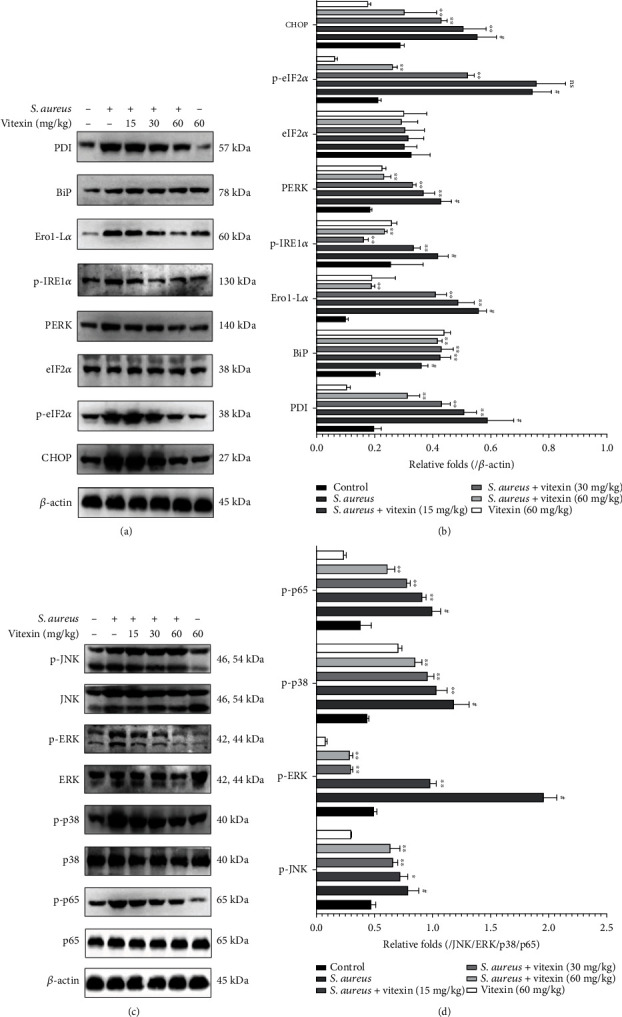
Vitexin relieved ER stress to inactivate MAPK and NF-*κ*B signaling pathways in *S. aureus*-induced mastitis on mice. (a) The protein levels of PDI, BiP, Ero1-L*α*, p-IRE1*α*, PERK, p-eIF2*α*, and CHOP in mammary gland tissues. (c) The expression levels of JNK, ERK, p38, and p65 proteins in mammary gland tissues. (b, d) Protein gray values were measured by the ImageJ software. Data was expressed as means ± SEM of three independent experiments. #*p* < 0.01 vs. the control group. ^∗^*p* < 0.05 vs. the *S. aureus* group; ^∗∗^*p* < 0.01 vs. the *S. aureus* group.

To explore the molecular mechanism of vitexin's antioxidant and anti-inflammatory effects, Western blot was used to study the protein expression of ER stress and MAPK/NF-*κ*B pathway. As displayed in Figures 9(a) and 9(b), the expression of BiP in the mouse breast tissue stimulated by *S. aureus* was significantly reduced compared to the control group, while PDI, Ero1-L*α*, p-IRE1*α*, PERK, and CHOP protein levels were restrained in the vitexin-treated mice with mastitis. Besides, p-JNK, p-ERK, p-p38, and p-p65 proteins were decreased in the *S. aureus* + vitexin group compared with the *S. aureus* group (Figures 9(c) and 9(d)).

## 4. Discussion

Dairy cow mastitis drastically reduces milk production and milk quality, posing a serious threat to the economic benefits of the dairy industry [25, 48]. The decrease in milk production during mastitis in dairy cows is due to pathogens (such as *S. aureus* and *Escherichia coli*) that induce hypoxia, oxidative stress, and apoptosis, while suppressing the expression of milk genes (Csn2, Lalba, and Csn1s1) in the mammary gland [49, 50]. Through the analysis of the GEO dataset (GSE94056 and GSE139612), we also found that *S. aureus* infection caused a large number of DEGs in the bovine mammary gland tissues and cells, which are inseparable from the activation of oxidative stress induced-ER stress and inflammation-related signaling pathways. Therefore, looking for Chinese herbal medicines that have both antioxidant and anti-inflammatory properties is very likely to become drugs for the treatment of mastitis.

Vitexin, a natural flavonoid compound found in many plant species [35], has been widely reported to have antioxidant, anti-inflammatory, antiviral, neuroprotective, and cardioprotective effects [27, 34]. However, few studies have explored the effect of vitexin on mastitis. In this study, in vivo and in vitro models of mastitis were successfully established. We found that vitexin reduced the production of ROS, protected the activity of antioxidant enzymes, and inhibited cell apoptosis and the production of proinflammatory cytokines in both MAC-T cell and mouse breast tissues. Previous studies have also confirmed that vitexin enhanced cell viability and/or decreased tissue damage by upregulating cell resistance to oxidative stress inducers [30, 31, 51]. Meanwhile, the anti-inflammatory activity of vitexin has attracted more and more attention, and there has been a lot of research involved. Vitexin downregulated the release of inflammatory cytokines (TNF-*α*, IL-1*β*, and IL-6) and enzymes (iNOS) by regulating transcription factors and kinases (Nrf-2, NF-*κ*B, and MAPKs) [52, 53]. To explore the molecular mechanism of vitexin's antioxidation and anti-inflammation, we focused on the connection between ER stress and inflammation.

As a nuclear receptor transcription factor, PPAR*γ* is involved in the regulation of ROS by regulating redox and biosynthetic processes in mitochondria [54, 55]. Activation of PPAR*γ* inhibited oxidative stress and also negatively regulated the expression of AP-1, NF-*κ*B, and other inflammatory response genes [56]. In the present study, PPAR*γ* was found through the intersection of prediction target genes of vitexin and inflammatory disease-related genes. The expression level of PPAR*γ* was suppressed during *S. aureus* infection, while vitexin treatment enhanced its protein expression. Moreover, GW1929 significantly hindered the increase of ROS in *S. aureus*-infected cells. GW9662 reversed vitexin-induced ROS inhibition under *S. aureus*-stimulated conditions. From these results, we speculated that vitexin might alleviate oxidative stress by regulating PPAR*γ*. Oxidative stress is caused by the excessive production of ROS, which interferes with the ER protein folding mechanism to result in the accumulation of abnormal proteins in the ER, thereby causing ER stress [16, 52]. Our study found that vitexin significantly inhibited PDI, Ero1-L*α*, p-IRE1*α*, PERK, and CHOP protein and promoted the expression of BiP in *S. aureus*-induced mastitis, suggesting that vitexin effectively prevented the production of ROS amplified by ER and programmed cell death triggered by CHOP.

ER stress is also involved in the regulation of immune signal pathways, such as MAPK and NF-*κ*B signaling pathways [57]. IRE1*α* can stimulate the activation of apoptosis signal kinase-1 (ASK1) and then activate downstream kinases Jun-N-terminal kinase (JNK) and p38 mitogen-activated protein kinase (p38 MAPK) [21, 58]. In this study, p-JNK, p-ERK, and p-p38 were significantly increased under *S. aureus* challenge. Vitexin treatment downregulated MAPK signaling proteins in the infected group compared with the untreated group. Therefore, we conjectured that vitexin might reduce the production of inflammatory factors by inhibiting the activation of MAPKs. PERK can also indirectly induce NF-*κ*B through eIF2*α* [59]. Vitexin inhibited p-p65 protein expression when *S. aureus* activated p65, indicating that vitexin alleviated inflammation and apoptosis partly by regulating the NF-*κ*B signaling pathway.

## 5. Conclusion

**Figure 10:**
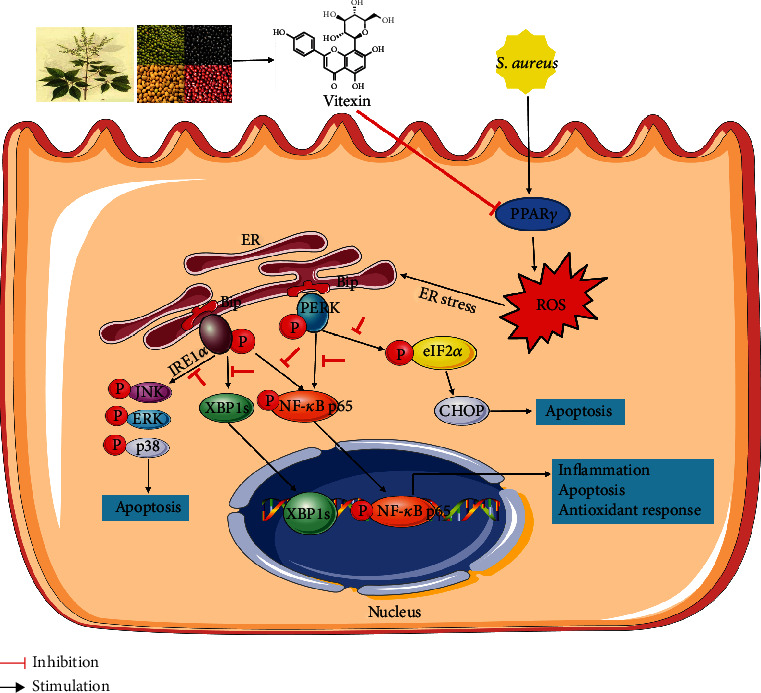
Schematic diagram of the therapeutic effect of vitexin on *Staphylococcus aureus*-induced mastitis. Vitexin inhibited the production of ROS by promoting PPAR*γ* activity, increased the activity of antioxidant enzymes, and reduced inflammatory cytokines and apoptosis by alleviating ER stress and inactivation MAPKs and NF-*κ*B signaling pathways.

In summary, *S. aureus*-induced mastitis produced a large number of DEGs, which were mainly involved in immune signaling pathways, apoptosis, and ER stress. Vitexin could inhibit the production of ROS by promoting PPAR*γ* activity, increased the activity of antioxidant enzymes, and reduced inflammatory cytokines and apoptosis, possibly by alleviating ER stress and inactivation MAPKs and NF-*κ*B signaling pathway (Figure 10). Vitexin may become a potential drug for the treatment of mastitis in dairy cows.

## Figures and Tables

**Table 1:** Primers used for qPCR.

Species	Gene name	Primer sequence (5′–3′)	GenBank accession number	Product size (bp)
Bos taurus	IL-1*β*	Sense: GGCAACCGTACCTGAACCCA Antisense: CCACGATGACCGACACCACC	NM_174093.1	206
	IL-6	Sense: ATGCTTCCAATCTGGGTTCA Antisense: GAGGATAATCTTTGCGTTCTTT	NM_173923.2	268
	TNF-*α*	Sense: ACGGGCTTTACCTCATCTACTCA Antisense: GGCTCTTGATGGCAGACAGG	NM_173966.3	141
	GAPDH	Sense: TGCTGGTGCTGAGTATGTGGTG Antisense: CAGTCTTCTGGGTGGCAGTGAT	NM_001034034.2	296

Mus musculus	IL-1*β*	Sense: TGCCACCTTTTGACAGTGATG Antisense: AAGGTCCACGGGAAAGACAC	NM_008361.4	220
	IL-6	Sense: TCTTGGGACTGATGCTGGTG Antisense: TTGCCATTGCACAACTCTTTTC	NM_031168.2	178
	TNF-*α*	Sense: GGTCCCCAAAGGGATGAGAAGT Antisense: TTGCTACGACGTGGGCTACA	NM_013693.3	124
	GAPDH	Sense: AAGAGGGATGCTGCCCTTAC Antisense: CCAATACGGCCAAATCCGTTC	NM_001289726.1	123

## Data Availability

The data used to support the findings of this study are available from the corresponding author upon request.

## Abbreviations

BiP: Immunoglobulin heavy chain binding protein
CHOP: C/EBP-homologous protein
DEGs: Differentially expressed genes
ER: Endoplasmic reticulum
Ero1-L*α*: ER-residing protein endoplasmic oxidoreductin-1 like L*α*
GO: Gene ontology
HPLC: High pressure liquid chromatography
IRE1*α*: Inositol-requiring enzyme 1*α*
JNK: Jun N-terminal kinase
KEGG: Kyoto Encyclopedia of Genes and Genomes
MAPK: Mitogen-activated protein kinase
MPO: Myeloperoxidase
NF-*κ*B: Nuclear factor-kappa B
PPI: Protein-protein interaction network
PPAR*γ*: Peroxisome proliferator-activated receptor
PDI: Protein disulfide isomerase
PERK: Protein kinase R- (PKR-) like endoplasmic reticulum kinase
ROS: Reactive oxygen species
*S. aureus*: *Staphylococcus aureus*
UPR: Unfolded protein response.
